# A Case Study of Upper-Room UVGI in Densely-Occupied Elementary Classrooms by Real-Time Fluorescent Bioaerosol Measurements

**DOI:** 10.3390/ijerph14010051

**Published:** 2017-01-08

**Authors:** Chunxiao Su, Josephine Lau, Fang Yu

**Affiliations:** 1 Durham School of Architecture Engineering and Construction, University of Nebraska-Lincoln, 1110 S 67th Street, Omaha, NE 68182, USA; csu@huskers.unl.edu; 2 Department of Biostatistics, College of Public Health, University of Nebraska Medical Center, 984375 Nebraska Medical Center, Omaha, NE 68198, USA; fangyu@unmc.edu

**Keywords:** ultraviolet germicidal irradiation, school, indoor air quality, real-time detection, fluorescent bioaerosol

## Abstract

Recently, the requirement to continuously collect bioaerosol samples using shorter response times has called for the use of real-time detection. The decreased cost of this technology makes it available for a wider application than military use, and makes it accessible to pharmaceutical and academic research. In this case study, real-time bioaerosol monitors (RBMs) were applied in elementary school classrooms—a densely occupied environment—along with upper-room ultraviolet germicidal irradiation (UVGI) devices. The classrooms were separated into a UVGI group and a non-UVGI control group. Fluorescent bioaerosol counts (FBCs) were monitored on 20 visiting days over a four-month period. The classroom with upper-room UVGI showed significantly lower concentrations of fine size (<3 μm) and total FBCs than the control classroom during 13 of the 20 visiting days. The results of the study indicate that the upper-room UVGI could be effective in reducing FBCs in the school environment, and RBMs may be applicable in reflecting the transient conditions of the classrooms due to the dynamic activity levels of the students and teachers.

## 1. Introduction

Bioaerosols are biological aerosol particles suspended in air. This subset of aerosols contains fungi, viruses, bacteria, spores, and pollen. Bioaerosols in indoor environments—especially at high concentration—have been associated over the last several decades with a wide range of adverse health effects, such as inflammatory, respiratory, and allergic reactions [1,2,3,4,5]. Research has been carried out in schools and childcare environments, which are usually densely occupied. When an increase in microbiological contaminants was identified together with excessive dampness, Bornehag [6] and Zuraimi [7] found that there were negative impacts on the attendance of students/children.

Upper-room ultraviolet germicidal irradiation (UVGI) with a wavelength range of 100–280 nm (UV-C) has been verified to efficiently disinfect airborne microbial organisms by inhibiting their ability to replicate [8,9,10,11,12,13]. In these research studies, *Bacillus atrophaeus* and *Escherichia coli* were nebulized. There are studies which found the concentration of indoor aerosols is positively associated with incidences of tuberculosis (TB), especially for people with a greater health risk [14,15]. UVGI has been proven to successfully reduce the spread of TB in high-risk settings [16,17]. The on-site performance of upper-room UVGI was evaluated by the transmission of tuberculosis to guinea pigs. The TB infection of the guinea pigs in the UVGI group was reduced to 9.5%, compared to 35% of those in the control group [18]. The methods of bioaerosol collections applied in the UVGI studies above were all based on traditional culturing and sampling methods, such as impactors and impingers. Alternatively, rapid detection of airborne microorganisms could provide a sufficient amount of data to draw a complete picture of transient bioaerosol levels over an extended time period. The upper limit of detection of fluorescence-based real-time bioaerosol monitoring devices could reach around 6 × 10^7^ number/m^3^ in the laboratory environment [19]. The devices had been tested on different species of airborne fungi under controlled laboratory conditions [20,21]. Additionally, limited tests with this technology have been undertaken in occupied indoor environments, including hospitals [22], university classrooms [23], and cleanroom areas [24,25]. The development of on-line techniques as real-time bioaerosol monitors (RBMs) has brought the possibility to evaluate the bioaerosol removal effects by air cleaning devices at proper time resolution and cost.

The objective of this study was to evaluate the effect of upper-room UVGI air cleaners on fluorescent bioaerosol counts (FBCs) in elementary classrooms. Based on our knowledge, it is one of the first studies to evaluate the performance of UVGI devices in a field study with RBMs on a broad range of fluorescent bioaerosols. By continuously monitoring fluorescent bioaerosol concentrations in UVGI and control classrooms, a detailed observation of the change in FBCs over time was obtained. A proper procedure for the application of RBMs in environments such as classrooms and data analysis is applied in the methodology, and the result is discussed.

## 2. Methodology

### 2.1. Location

The public elementary school tested is located in the U.S. Midwest. Two classrooms (one used for reading and one for math) with the same floor area of 85 m^2^ and with the same class schedules were selected. The class sizes were between 25 and 30 students. Each classroom has a separate ventilation system with no shared recirculation pathways.

### 2.2. Experiment Design and Procedures

**Figure 1:**
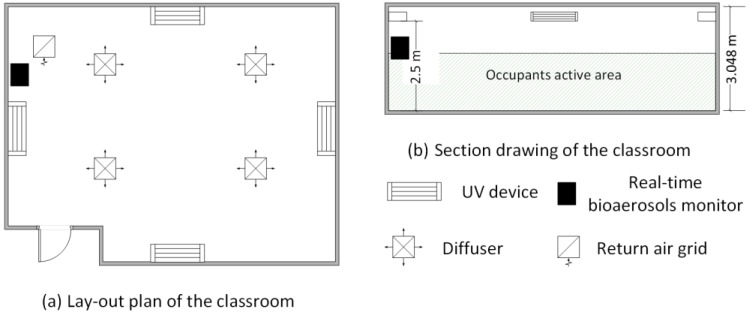
Floor plan of the classroom and location of the real-time bioaerosol monitors (RBMs) (**a**) Lay-out plan of the tested classrooms with locations of UV devices and RBM; (**b**) Section drawing of the tested classrooms showing the heights of UV devices and RBM. UV: ultraviolet.

For the study, one classroom was installed with four units of upper-room UVGI air cleaners and the other one was set as the non-UVGI control room. Samples of fluorescent bioaerosols were collected and measured over a four-month period from October 2012 to January 2013. Two RBMs were placed in each of the tested classrooms. The sampling points were located close to the returning air grille because the air would be well-mixed at the returning grille as compared to other spaces in the classrooms [26]. Figure 1 shows the location of the RBMs in each classroom.

The RBMs were placed in the classrooms prior to the students’ entry to ensure that the FBCs would start recording before the room was occupied. The fluorescent bioaerosols were continuously monitored through an entire day until one hour after student dismissal. In October and November, the classrooms were monitored for one day in each month. In December and January, the RBMs were placed in the classrooms for two weeks to collect data for multiple days.

There are four supply air inlets and one exhaust air outlet in each classroom. The air flow rates of inlets and outlets were tested by an ALONOR air flow hood (TSI, Inc., Shoreview, MN, USA). Temperature and relative humidity were measured monthly by an OMEGA OM-73 (OMEGA Engineering Inc., Stamford, CT, USA) temperature/humidity data logger. The RBM tests were carried out monthly, concurrently with the traditional culture-based method. Upper-room UVGI units were installed in two selected classrooms. In each classroom, four UVGI units (Lumalier WM-136, Lumalier Corporation, Memphis, TN, USA), each with a 36 W ultraviolet (UV) lamp, were installed on four walls as shown in Figure 1a. The UVGI units were installed above 2.4 m in height to fulfill the safety requirement for occupants (0.2 μw/cm^2^) [27]. A radiometer (Model IL 1700A with SED 240 detector, International Light Inc., Newburyport, MA, USA) was used to measure the UV irradiance of upper-room UVGI units in the field. The UV irradiance measurements were discussed in a previous study [28]. The UV lamps ran continuously during both occupied and unoccupied times, and were replaced after 8000 running hours.

### 2.3. Fluorescent Bioaerosols

The RBM model used in this study was the IMD-A series manufactured by BioVigilant. (BioVigilant Systems Inc., Tucson, AZ, USA).

A complete RBM system includes two main components: one particle size detector determining the size of each individual particle, and one fluorescence detector deciding if the particle is biological or inert by the presence or absence of intrinsic fluorescence. Inside the monitors, UV illumination is used to concurrently examine each particle for the presence of the metabolites nicotinamide adenine dinucleotide (NADH) and riboflavin, which are necessary intermediates for the metabolism of a living organism and therefore exist in microbes such as bacteria and fungi [22,23]. The system can detect and quantify particles within a range of 0.5–10 μm. The RBMs categorizes the particles into three groups: biological, inert, and total particles. The biological particle is the same as the fluorescent bioaerosol used in this paper. The number of total aerosols is a parameter directly measured by the particle size detector inside the RBM, similar to a reading of an optical counter that reflects the total number of aerosols. The number of inert aerosols is calculated by subtracting the FBCs from the total particles.

The RBMs kept the flow rate constant at 1.15 L/min, which ensured that the sampling pumps in the devices ran quietly and would not distract the occupants from daily activities. The data was stored on an 80 GB hard drive, which fulfills the requirement for a long running time. The unit of concentration was converted to number per cubic meters (number/m^3^).

### 2.4. Data Analysis

RBMs record three categories of aerosols: bioaerosol, inert aerosol, and total aerosol. The three categories were obtained in six channels from size 0.5 μm to 10 μm. These channels were grouped into several ranges. The fluorescent bioaerosols from 0.5–3 μm were summarized as fine size A (<3 μm), 0.5–7 μm as fine size B (<7 μm), and the 10 μm and above as coarse size. Fine size A is the size range that dominated in the classroom collected by the RBM. Fine size B is comparable to the size range of bioaerosol samples by the second stage of the two-stage Andersen samplers.

**Figure 2:**
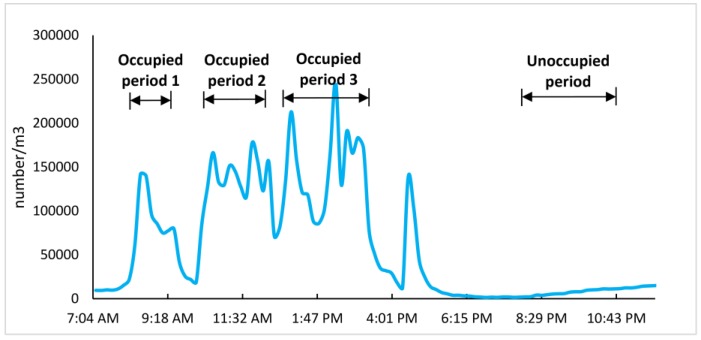
The figure shows a selection of occupied periods fluorescent bioaerosol counts (FBCs) at fine size A (0.5–3 μm) in the non-ultraviolet germicidal irradiation (UVGI) classroom from a typical sampling day.

In this study, the data were divided into two time periods: occupied and unoccupied time. The analysis focused on the data collected from occupied time. The occupied periods were picked as 08:20–09:20, 10:10–12:00, and 12:45–15:15, which matched with the class schedules for both the UVGI and control classrooms. A curve of continuously measured bioaerosols during one visiting day is shown in Figure 2 to demonstrate the three occupied periods.

To reflect the aerosol generated by occupant-related activities, an average concentration during the unoccupied period was subtracted from the occupied concentrations at each data point. This adjustment helps to minimize the influence of classroom settings on the samples. The unoccupied period was selected as from 20:00 to 23:00 for each visiting day. Since the RBMs were not monitoring the classrooms for a 24 h period during the first two months, only the data from the latter two months are shown in this paper. The mean of concentrations during the unoccupied period was calculated as ${\bar{C}}_{unoccupied}$. Starting with each data point from the occupied period, $C_{occupied,t}$, and subtracting the mean number of the unoccupied period, ${\bar{C}}_{unoccupied}$, obtains a new data for the same time point, $C_{occupied,t}^{\prime }$, as shown in Equation (1). This procedure of adding adjustment was also applied to the fractions of fluorescent bioaerosols to total aerosol.

(1) $$C_{occupied,t}^{\prime }=C_{occupied,t}-{\bar{C}}_{unoccupied}$$

The raw data were first reorganized in Excel 2013 (Microsoft Inc. Redmond, WA, USA), then averaged as 1-min interval data to reduce the number of data points to 1/6 of the original amount. The non-parametric Wilcoxon signed ranks test was applied to determine if differences in concentrations existed between UVGI and non-UVGI control classrooms. SPSS version 21 (IBM Corporation, Armonk, NY, USA) was used to conduct the statistical analysis.

## 3. Result

The results of FBCs measurement in the classrooms are presented in the following paragraphs. The effect of the unoccupied background adjustment was also explored.

### 3.1. Environmental Parameters

The average indoor temperature and relative humidity for the UVGI and non-UVGI control classrooms were well-maintained during the measurement. The indoor temperatures fell to a range of 22.3 °C to 22.7 °C, and the range of relative humidity was 24.1% to 34.6% throughout the entire testing period. Based on the results of ventilation measurement, the supply air rates and return air rates were stable throughout the sampling. The supply air and return air rates for the UVGI classroom were 0.47 m^3^/s and 0.38 m^3^/s, respectively. For the control classroom, the supply air and return air rates were 0.45 m^3^/s and 0.38 m^3^/s, respectively. The measurement error of the flow hood was ±0.017 m^3^/s.

### 3.2. Concentrations before Adjustment to Background

The FBCs throughout all twenty visiting days are presented in Table 1. The raw concentrations of fine size A fluorescent bioaerosols were usually below 300,000 number/m^3^. For the fluorescent bioaerosols of coarse size (10 μm), the concentrations were generally lower than 100,000 number/m^3^.

**Figure 3:**
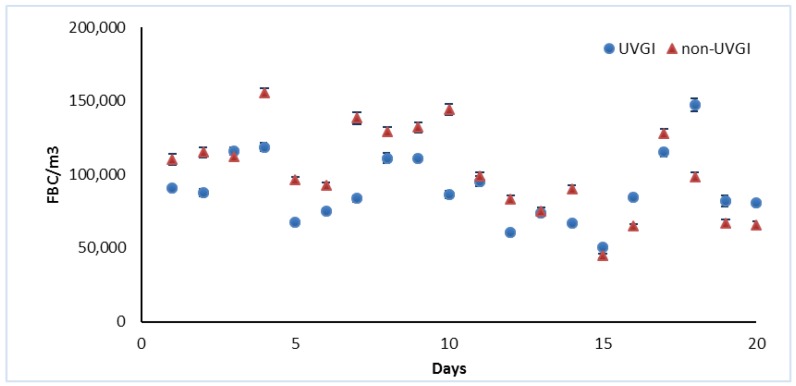
Comparison of fine size A FBCs between UVGI and non-UVGI control classrooms, before adjustment. non-UVGI: non-UVGI classrooms.

The average FBCs from the non-UVGI control classroom for 12 visiting days were significantly higher than those from the UVGI-control classroom (Figure 3). The UVGI and non-UVGI classrooms were compared for each visiting day. The statistical tests show a significant difference between two classrooms (*p*-values < 0.05). The only exceptions are Day 11 and 13, which showed a slightly lower concentration in the UVGI classroom than the non-UVGI control classroom, with no statistically significant differences (*p*-value > 0.05).

For coarse size bioaerosols (10 μm), fifty percent of the visiting days showed lower levels of FBCs in the UVGI classroom, distributed seemingly randomly throughout the entire study. The total FBCs (0.5 μm–10 μm), however, shows the same results as the fine size A and B. This is because the smaller size FBCs always dominated the overall count.

### 3.3. Concentrations after Adjustment to Background

The adjustment was made as stated in the data analysis portion of the methodology Section 2.4 by Equation (1) before comparing the UVGI and non-UVGI classrooms. The newly calculated values reflected the increased bioaerosols due to occupants and their activities. After this background adjustment was calculated, the FBCs were reduced to a range below 250,000 number/m^3^. The coarse size FBCs dropped to a range with an upper limit of 30,000 number/m^3^. This adjustment indicates that the level of FBCs during occupied times was always higher than unoccupied times. This phenomenon existed in both UVGI and non-UVGI classrooms.

## 4. Discussion

### 4.1. Comparing to Cultural Method

There are relatively few studies utilizing fluorescent bioaerosol monitors in the literature. In laboratory studies, the concentration of released bioaerosols in indoor environments reached the highest level of 10,000,000 number/m^3^ [29]. Agranovski et al. [19] studied the relationship between the counting efficiency of a fluorescence-based device and aerosol concentrations. They found that fluorescent aerosol readings were reduced with an increase of total aerosol concentration around up to 10 × 10^6^ number/m^3^. The same model of RBMs used in our study has been tested in hospital environments [22]. Results have recorded the greatest concentrations at around 200,000 number/m^3^. The readings of RBMs from six hospitals agreed with the culture-based samples. Significant correlations between the culture-based concentrations and FBCs by RBMs were observed. Dalmaso [29] performed tests with both six-stage Andersen samplers and RBMs in a laboratory environment. In that study, the highest level of bioaerosol recorded was below 300,000 number/m^3^, and the two sampling methods showed the same variability in the concentrations of bioaerosols.

**Figure 4:**
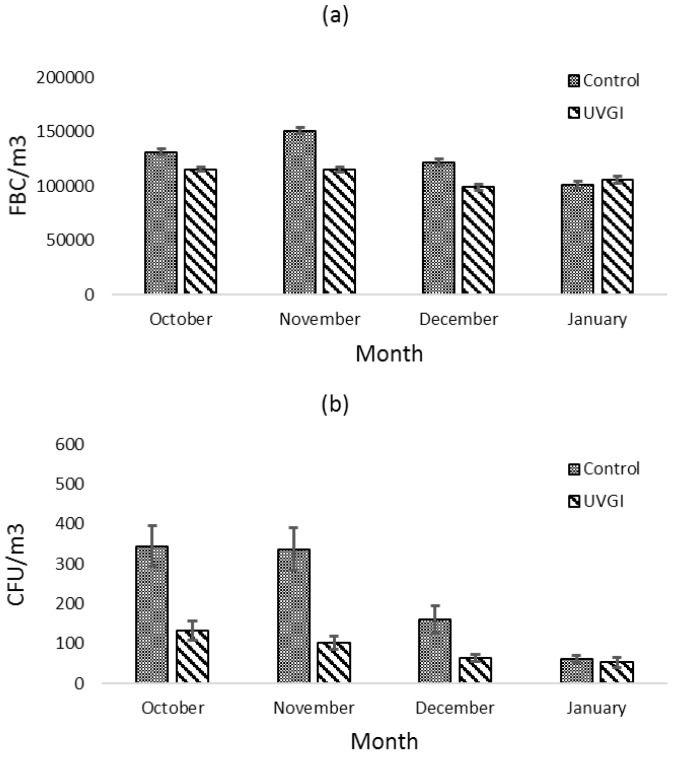
Comparison of results from two bioaerosols sampling methods (**a**) Monthly FBCs RBMs at fine size B (<7 μm) in UVGI and non-UVGI control classrooms before the adjustment; (**b**) The monthly concentration of airborne culturable bacteria using Andersen impactor at the fine size (<8 μm) in the UVGI and non-UVGI control classrooms. CFU: colony forming units.

This study indicates that RBMs detected fewer FBCs in the UVGI classroom compared to the non-UVGI control room during the four months of measurement. Besides RBMs, another measurement of bioaerosols utilizing two-stage Andersen impactors to collect airborne culturable bacteria at 0.8 μm–8 μm and >8 μm was reported in a previous study [28]. In that experiment, the cultured samples were collected within one to two hours following student dismissal. The data were separated by month to compare the results of the two sampling methods. Figure 4a shows the results of the fine size A FBCs. Figure 4b shows the results of the traditional culture-based method. The culture-based sampling method had the same statistical results as the RBMs when comparing bioaerosol concentrations in the UVGI and non-UVGI control classrooms.

Both the RBMs and the traditional method found that the fine size B FBCs (<7 μm) in the UVGI classroom were statistically significantly lower than the non-UVGI control classrooms during the first 3 months (*p*-values < 0.05). The RBMs detected higher peak concentrations (20,000 number/m^3^) for fine size B FBCs than the readings from the two-stage Andersen samplers (600 CFU/m^3^). One possible reason is that the microbial population in the field could be highly heterogeneous. The cultural method may significantly underestimate the total concentration of viable microorganisms due to the bacterial competitiveness during cultivation [30]. There are studies that found that only a small proportion of microorganisms will grow on culture agars [31], raising the concern that using the cultural method could significantly underestimate the actual concentration. In addition, the significant time requirement of culture-based methods may limit researchers from detailed observation of bioaerosols [32,33,34]. Thus, continuous observations cannot be made quickly. The traditional method also requires extensive labor time to obtain the data, which may not work well when sampling an environment requires rapid response and decision making.

### 4.2. Effect of Adjustment

Although the selected classrooms shared the same floor plan, ventilation system, and class schedule, other (albeit difficult to observe) differences could exist in the classroom settings to create different concentration levels during the unoccupied conditions (as the background difference). The adjustment to occupied time data by unoccupied data must consider both the difference and timing issues. By adding this adjustment, data points from the same time were paired between the UVGI and non-UVGI control classrooms.

When comparing the concentrations before and after the adjustment, there were the same number of visiting days showing a statistical significance. The same conclusion can be made for both fine size A and B FBCs. This may indicate that the adjustment has eliminated part of the differences resulting from environmental factors other than occupants, and could help reduce the bias of comparing two different classrooms in the field.

### 4.3. Occupants and Their Activities Cause the Increase of FBCs (Reflect the Difference between Occupied and Unoccupied Periods)

It has been found that occupants and their activities are a main source of bioaerosols in indoor environments [35,36]. High concentration of environmental bioaerosols raised by occupants may be related to a higher risk of airborne infectious diseases with patients at present. Another study with fluorescence-based devices also revealed significant differences of bioaerosol concentrations between the occupied and unoccupied periods in university classrooms [23]. This usually raises public health concerns. So, quick detection of indoor bioaerosols during occupied times in buildings has significant practical meaning. In our study, airborne culturable bacteria were collected by the culture-based method, and the samples were obtained immediately after the occupied time. The result might not represent the actual bioaerosols concentration in the occupied time. In this measurement by RBMs, the sampling period covered both occupied and unoccupied periods. However, due to the operating procedure and noise level of the impactors, agar plates could only collect bioaerosols samples right after the occupied time, but not exactly at the occupied time. The RBMs filled this gap and provided a complete data map through all visiting days. In addition, RBMs obtained data exactly from the occupied time, which can more precisely depict the variability of FBCs.

### 4.4. Scope and Limitations

The scope of this field case study is to find the reduction in bioaerosols by upper-room UVGI with a rapid detection technique such as RBMs. Although lower concentration of FBCs in the UV-irradiated classroom were successfully measured, there are several limitations to this study.

The comparison of the concentrations is based on the assumption that the student activities in both UVGI and non-UVGI control classrooms were within the same levels. Though the number of students in two classrooms was very close, the influence on the data generated by undistinguished student activities was difficult to determine. We also did not closely monitor the health conditions of the students due to the limited of timeframe and budget of this study. Our study focuses on the reduction of FBCs by upper-room UVGI to lower the potential risk on occupants’ health in indoor environments such as a classroom. For occupants like elementary students, further effort will be needed to explore the relationship between the development of immune systems and natural bioaerosol load, regarding the hygiene hypothesis developed in recent decades which states the necessity of early childhood exposure to microorganisms [37].

In addition, there are experimental results that have shown that fungal bioaerosols inactivated by sub-seconds exposure to thermal energy can still produce fluorescence signals [38]. There is no available research about how UV light affects bioaerosols that generate a fluorescence signal. In one study by Agranovski et al. which compared a fluorescent device to culture-based methods measuring three types of airborne bacteria, laboratory tests found the physiological condition of bacteria—specifically injured cells—was below the sensitivity level of a specific fluorescent device [30]. This indicates that the amount of fluorophores within injured bacteria may not trigger the fluorescent signal, making them undetected by an RBM.

## 5. Conclusions

This study evaluated the reduction of fluorescent bioaerosols counts by upper-room UVGI in classrooms with RBMs, and came to the following conclusions:

1. Upper-room UVGI devices can reduce the fluorescent bioaerosols found in an elementary classroom. Daily samples collected through a four-month study showed that average concentrations of fine size (<3 μm) FBCs in 12 visiting days from the control classroom were statistically significantly higher than those from the classroom equipped with UVGI devices. The RBMs provided statistically similar results on the performance of upper-room UVGI devices when comparing to a parallel study using the traditional culture-based sampling method.
2. With the RBMs, both FBCs and total aerosols were monitored. Comparing the concentrations from occupied and unoccupied periods found significantly higher FBCs during the occupied periods of all visiting days. This result supports the notion that humans and their activities are the primary cause of an increase of detectable FBCs during occupied periods.

## Figures and Tables

**Table 1:** Daily average fluorescent bioaerosol count (FBC) (**a**) fine size A (<3 μm), and (**b**) coarse size (10 μm).

**Month**	**Day**	**UVGI**	**SD**	**Control**	**SD**	***p*-Values**	**Proportion (%) of Reduction**
**Table 1a**							
**October**	DAY1	115,507	37,662	130,675	68,453	***<0.001***	***11.6%***
**November**	DAY2	114,829	42,628	150,561	63,257	***<0.001***	***23.7%***
**December**	DAY3	124,344	40,464	115,959	45,918	<0.001	−7.2%
	DAY4	123,349	44,746	147,970	45,798	***<0.001***	***16.6%***
	DAY5	72,802	30,987	104,317	34,547	***<0.001***	***30.2%***
	DAY6	82,625	28,117	101,161	39,277	***<0.001***	***18.3%***
	DAY7	74,993	44,155	111,091	77,674	***<0.001***	***32.5%***
	DAY8	118,451	60,586	139,756	49,941	***<0.001***	***15.2%***
	DAY9	119,348	46,224	139,737	65,215	***<0.001***	***14.6%***
	DAY10	93,896	44,664	156,205	64,377	***<0.001***	***39.9%***
	DAY11	81,260	56,607	81,455	51,393	**0.747**	**0.2%**
**January**	DAY12	91,892	27,630	106,601	43,190	***<0.001***	***13.8%***
	DAY13	74,925	30,986	77,691	46,437	**0.293**	**3.6%**
	DAY14	78,767	26,301	104,253	48,711	***<0.001***	***24.4%***
	DAY15	63,986	20,363	59,964	24,181	<0.001	−6.7%
	DAY16	92,164	36,499	70,473	31,362	<0.001	−30.8%
	DAY17	119,133	55,323	135,855	50,214	***<0.001***	***12.3%***
	DAY18	159,660	79,687	106,932	50,690	<0.001	−49.3%
	DAY19	115,687	67,385	96,060	45,830	<0.001	−20.4%
	DAY20	72,978	40,714	53,381	39,227	<0.001	−36.7%
**Table 1b**							
**Month**	**Day**	**UVGI**	**SD**	**Control**	**SD**	***p*-Values**	**Proportion (%) of Reduction**
**October**	DAY1	1812	1020	2539	1604	***<0.001***	***28.6%***
**November**	DAY2	1731	997	2073	1279	***<0.001***	***16.5%***
**December**	DAY3	2058	1133	1911	989	0.034	−7.7%
	DAY4	2408	1433	2361	1223	<0.001	−2.0%
	DAY5	1406	1031	1521	843	***0.008***	***7.6%***
	DAY6	1437	947	1596	935	***0.002***	***10.0%***
	DAY7	1338	1232	1341	1229	*0.584*	0.2%
	DAY8	1872	1401	1735	852	0.732	−7.9%
	DAY9	2087	1230	1432	1029	<0.001	−45.8%
	DAY10	1352	1189	1940	1284	***<0.001***	***30.4%***
	DAY11	1431	1361	977	1016	<0.001	−46.4%
**January**	DAY12	1446	830	1612	936	***0.002***	***10.3%***
	DAY13	1419	1121	1896	2012	***<0.001***	***25.2%***
	DAY14	1751	1252	2045	1459	***0.001***	***14.4%***
	DAY15	901	496	997	738	**0.092**	**9.6%**
	DAY16	1121	735	999	614	0.021	−12.3%
	DAY17	1507	1061	1559	820	**0.144**	**3.3%**
	DAY18	1500	960	1260	841	<0.001	−19.0%
	DAY19	1228	944	1140	810	0.306	−7.7%
	DAY20	885	681	587	585	<0.001	−50.7%

Bold numbers indicate lower FBC in the ultraviolet germicidal irradiation (UVGI) room; Italic numbers indicate statistically significant higher FBC in the UVGI room; SD: standard deviation.
